# Optimization of HS-SPME-GC/MS Analysis of Wine Volatiles Supported by Chemometrics for the Aroma Profiling of *Trebbiano d’Abruzzo* and *Pecorino* White Wines Produced in Abruzzo (Italy)

**DOI:** 10.3390/molecules28041534

**Published:** 2023-02-05

**Authors:** Leucio Rossi, Martina Foschi, Alessandra Biancolillo, Maria Anna Maggi, Angelo Antonio D’Archivio

**Affiliations:** 1 Department of Physical and Chemical Sciences, University of L’Aquila, Via Vetoio, 67010 Coppito, Italy; 2 Hortus Novus, Via Campo Sportivo 2, 67050 L’Aquila, Italy

**Keywords:** wine aroma, volatile profile, solid-phase microextraction, gas-chromatography, experimental design, response surface, Trebbiano d’Abruzzo, Pecorino

## Abstract

Headspace Solid-Phase Microextraction coupled to Gas-Chromatography with Mass Spectrometry detection (HS-SPME/GC-MS) has been widely used to analyze the composition of wine aroma. This technique was here applied to investigate the volatile profile of Trebbiano d’Abruzzo and Pecorino white wines produced in Abruzzo (Italy). Optimization of SPME conditions was conducted by Design of Experiments combined with Response Surface Methodology. We investigated the influence of the kind of sorbent, PDMS, CW/DVB, or PDMS/CAR/DVB, and the effect of the fiber exposure time, temperature, and salt concentration on the total area of the chromatogram and the extraction efficiency of ethyl decanoate and 3-methyl-1-butanol, representative of apolar and polar compounds, respectively. The PDMS/CAR/DVB sorbent allowed the extraction of about 70 compounds, whereas only a part of these substances could be extracted on the PDMS and CW/DVB fibers. Reliable response surfaces for the total area and peak areas of the selected volatiles collected on the PDMS and PDMS/CAR/DVB sorbents and, in the latter case, principal component analysis were evaluated to find the optimal conditions. The optimized extraction conditions were applied for a preliminary comparison of the volatile profile of the two wine varieties and in a successive varietal discrimination study based on data-fusion approaches.

## 1. Introduction

Wine aroma originates from an intricate combination of hundreds of volatile organic compounds (mainly alcohols, esters, organic acids, aldehydes, ketones, and terpenes) already present in the grapes or formed in the fermentation and maturation processes [1]. Among the organoleptic characteristics, aroma is one of the most important to define wine quality and character, and can determine consumer acceptance. The volatile profile is influenced by ripeness, grape variety, geographical factors (climate and soil), wine-making practices (pH, temperature, and yeast activity in fermentation and aging conditions), oxidation, and wine defects [2,3,4]. Knowledge of the aroma composition is, therefore, an important source of information on wine quality and can be useful for the prediction of sensory properties [5,6], for geographical or varietal discrimination [7,8,9], and to guide the setup of production technologies aimed at exalting one or more aromas in the final wines [10,11,12].

Solid-phase microextraction (SPME) is a solvent-free, rapid, sensitive, reproducible, and easy-to-use method extensively applied for the concentration of the wine aroma components [7,13,14,15]. SPME is based on the sorption of the analytes on a fiber coating either put in contact with a gaseous phase or submersed in a liquid sample [16]. The analytes adsorbed and/or absorbed on the fiber (depending on the coating) can be successively analyzed by thermal desorption in the injection port of a conventional gas chromatography. Apart from the retentive properties of the fiber coating, several other factors (extraction time and temperature, ionic strength, sample volume, fiber conditioning, and desorption conditions) affect the extraction efficiency. Because of the intrinsically multivariate nature of SPME, identification of the optimal experimental conditions for the sample treatment by usual one-factor-at-a-time approaches, which do not consider possible interaction between factors, can be inefficient with respect to the use of time and resources, and may lead to suboptimal performances [17,18,19]. Multivariate methods, on the other hand, can provide fast, efficient, and cost-effective approaches for optimization purposes. Design of Experiments (DoE), in particular, allows for the identification of a relatively small number but informative experiments to be carried out in ad hoc designed combinations of the factors [17,18,20]. The experimental data collected in the DoE points can then be handled by the Response Surface Methodology (RSM) to model the multivariate dependence of the response from the factors and to predict the response value within the whole experimental domain of interest. DoE-RSM approaches in the development of SPME-based analysis of wines have been previously used for volatilomic characterization [15,21] and for the determination of specific classes of odorants [22,23], volatile compounds responsible for off-aromas [24,25], and pesticides [26]. 

In this study, we applied a headspace (HS)-SPME method coupled to gas chromatography-mass spectrometry (GC-MS) for the analysis of the aroma profile of two white wines produced in the Abruzzo region (central Italy), *Trebbiano d’Abruzzo* (TRE), and *Pecorino* (PEC). TRE is the major white wine produced in this region, obtained by *Trebbiano d’Abruzzo* and *Trebbiano Toscano* grapes extensively cultivated in the hills of the coastal territory and some internal areas [27]. PEC cultivars, which belong to the family of *Trebbiano* grapes as well, although cultivated since Roman times, had been replaced by the more productive *Trebbiano d’Abruzzo* variety, and it was thought extinct by the mid-20^th^ century. The rediscovery of *Pecorino* started in the 1980s, when some cuttings were taken from growing wild plants found in the bordering Marche region and re-implanted. Since then, the cultivation of the *Pecorino* variety in the Abruzzo and Marche regions has exponentially grown. Today, the most prestigious vineries of the Abruzzo region cultivate both PEC and TRE grapes that are used to produce mono-varietal wines. The main target of this investigation is the development of a method for the determination of the volatile profile of these two wine varieties. Actually, the present study was the preliminary step of a wider investigation, recently published [28], aimed at the multi-analytical characterization of TRE and other ancient wine varieties of the Abruzzo region that have been recently rediscovered, including PEC. The final goal of the multi-analytical characterization of the wine varieties was the identification of chemical markers useful for the varietal discrimination and authentication of mono-varietal wines. In this context, the quantitative determination of volatile components is not required, since the chromatographic profiles collected under pre-fixed experimental conditions, properly handled by chemometrics, are suitable for traceability purposes. Nevertheless, the volatile profiles collected by HS-SPME/GC-MS should be as informative both in terms of global intensity of the chromatogram and the number of chemical species providing a detectable signal. Bearing this in mind, the present work mainly focused on the identification of the optimal conditions of an SPME-based method for the extraction of the aroma volatiles of TRE and PEC wines. A preliminary comparison of the volatile profiles of the two wine varieties was made here for a limited number of samples and without taking care of the representativeness of the analyzed wines with respect to the geographical location of the production sites within the regional territory. This issue has been addressed in the successive development of the present investigation. To optimize the HS-SPME method, we explored the performance of three different fibers (PDMS, CW/DVB, and DVB/CAR/PDMS) and evaluated the influence of the exposure time of the sorbent to the sample headspace, sample temperature, and concentration of added salt on the extraction efficiency. DoE was applied to identify proper combinations of the above variables, while statistical treatment of the GC data was performed, when possible, by RSM. The DoE-RSM strategy was primarily applied to model the total intensity of the GC chromatogram with the aim of maximizing chemical information on the aroma profile of the wines. However, it is known that the partition coefficients of individual components both between the liquid sample and the headspace and between the headspace and the fiber are dependent on the analyte properties (solubility, polarity, volatility, among the others) [16]. Therefore, the relative contribution of individual volatiles to the observed GC/MS chromatograms can be dependent on the SPME conditions. For this reason, we also investigated the influence of the experimental conditions on the extraction of two selected compounds, ethyl decanoate and 3-methy-1-butanol, representing aroma components with different chemical structures and physico-chemical (solubility and volatility) properties. In addition, principal component analysis (PCA) was applied to the GC chromatograms collected with the selected fiber to investigate the effect of the SPME conditions on the composition of the aroma profile in more detail. Finally, a preliminary comparative evaluation of TRE and PEC aroma collected under the optimal HS-SPME conditions was accomplished. 

## 2. Results 

### 2.1. Qualitative Composition of Wine Aroma by HS-SPME-GC/MS

The qualitative identification of volatile components of TRE and PEC aroma was performed by GC/MS analysis by comparing both retention indices (RIs, collected in Table S1 available as Supplementary Material) and mass spectral patterns with the data reported in the literature and in the NIST14 library [29]. Using the three different fibers under the various experimental conditions defined by the DoEs adopted in the respective cases, we detected and identified more than 70 volatile components (listed in Table 1). The compounds found in the wine aroma belong to different chemical classes, including esters, organic acids, alcohols, aldehydes, and ketones. It can be observed that the number and variety of the detected volatiles are dependent on the fiber coatings. In particular, the DVB/CAR/PDMS fiber, which combines three stationary phases with different adsorbtion/absorption properties, can extract the maximum number of volatile components from the wine headspace, whereas the compounds extracted by the PDMS and CW/DVB fibers are lower in number and are mainly esters, followed by alcohols and organic acids. 

### 2.2. Optimization of HS-SPME on the PDMS Fiber

To maximize chemical information on the volatile profile of the wine aroma, we primarily investigated, by DoE-RSM, the influence of the SPME conditions on the total area of the GC/MS chromatogram, A_TOT_. This choice identifies a unique set of optimum conditions for the extraction of all the target analytes. All of the experiments were performed on the same wine sample belonging to the TRE variety. In the investigation of the performance of the PDMS fiber, the combinations of the exposure time (t) of the fiber to the headspace, the sample temperature (T), and the percentage (*w/v*) of NaCl (%NaCl) were defined according to a Box-Behnken DoE [20] with duplicate experiments in the central point. To evaluate the dependence of the fiber coating and SPME conditions on the extraction recovery of specific wine aroma components in more detail, we also modeled, by DoE-RSM, the dependence of the peak-area of two selected compounds, ethyl decanoate (ED) and 3-methyl-1-butanol (MB), from the SPME conditions. These two compounds, which can represent apolar and polar volatile components, respectively, were selected because they provide relative intense chromatographic peaks within the whole experimental domain. This condition minimizes experimental error in the collection of model responses.

**Figure 1:**
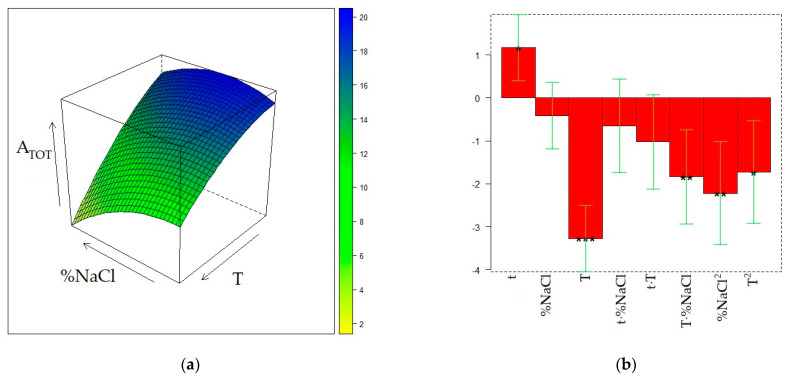
Optimization of HS-SPME profiling of wine volatiles extracted with the PDMS fiber: (**a**) response surface for the total area of the GC chromatogram (A_TOT_) at t = 30 min; (**b**) coefficients of the surface model; *p* < 0.05 (*), *p* < 0.01(**), *p* < 0.001 (***).

**Figure 2:**
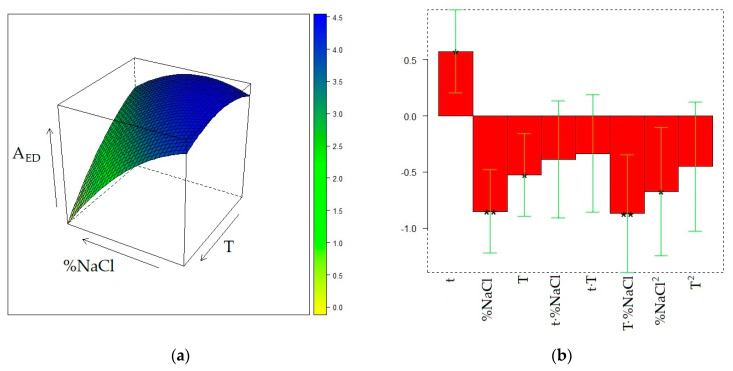
Optimization of HS-SPME profiling of wine volatiles extracted with the PDMS fiber: (**a**) response surface for the peak area of ethyl decanoate (A_ED_) at t = 30 min; (**b**) coefficients of the surface model; *p* < 0.05 (*), *p* < 0.01(**).

**Figure 3:**
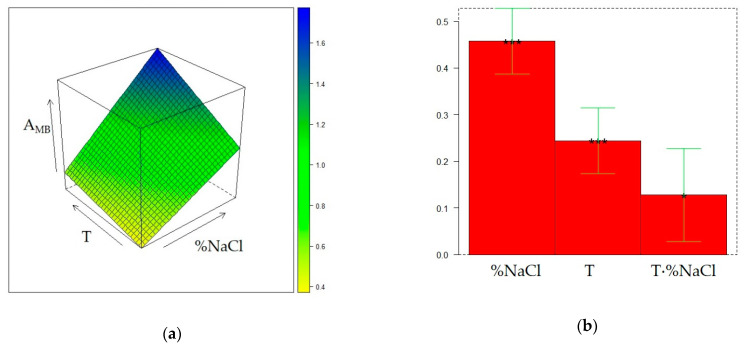
Optimization of HS-SPME collection of wine volatile profiles with the PDMS fiber: (**a**) response surface for the peak area of 3-methyl-1-butanol (A_MB_); (**b**) coefficients of the surface model; *p* < 0.05 (*), *p* < 0.001(***).

The matrix of the experiments and the experimental responses are displayed in Table 2. The surface models for the three responses were generated by quadratic regression after transformation of the values of the three factors (t, T, and %NaCl) into the corresponding levels (−1, 0, or 1). The final surfaces and the model coefficients for A_TOT_, A_ED_, and A_MB_ responses are displayed in Figure 1, Figure 2, and Figure 3, respectively, whereas the calculated responses in the DoE points are given in Table 2. The reliability of the surface models was also evaluated by the adjusted coefficients of determination in fitting and leave-one-out cross-validation (R^2^_adj_ and Q^2^_adj_, respectively) and by analysis of variance (ANOVA) [20], reported in Table S2 (Supplementary Material). 

Figure 1a displays the dependence of A_TOT_ from T and %NaCl, t being fixed at its maximum level (30 min). Since the t − -T and t-%NaCl interactions are non-significant, the shape of this surface does not depend on the exposure time of the fiber to the sample headspace, although the response decreases when t decreases because of the positive value of the t coefficient and non-significance of the t^2^ term (Figure 1b). The maximum for A_TOT_ can be found at t = 30 min, T = 30 °C, and %NaCl = 20. Starting from the 0 level for %NaCl (20%), a moderate A_TOT_ decrease is observed when %NaCl both rises and decreases, whereas the total area of the chromatogram dramatically decreases when the temperature grows, regardless of the NaCl content in the sample. The surface model developed for A_ED_ (Figure 2) results are significant (*p* = 0.0064) according to ANOVA and exhibit a relatively high R^2^_adj_ value (0.8767), but provides a noticeably worse predictive performance (Q^2^_ad j_= 0.5625) and a significant lack-of-fit (*p* = 0.035). In this regard, it must be noted that the T^2^ term, despite being non-significant, was anyway retained in the surface model because its elimination resulted in further worsening of the Q^2^_adj_ value. Nevertheless, the so-obtained surface model, although it may be unsuitable for quantitative prediction of the response, can be accurate enough to describe the qualitative trend of A_ED_ within the experimental domain. It can be noted that the shape of the A_ED_ surface is not much dissimilar from that of A_TOT_, but the maximum of the first response is slightly shifted toward the center of the experimental domain (T = 37 °C and %NaCl = 13). The similarity between the two surfaces is not unexpected because ED is one of the volatile components contributing the most to the wine volatilome. In this regard, it must be noted that the relative contribution of the ED peak area to the total area of the chromatogram noticeably increases at the maximum temperature and minimum NaCl concentration.

Development of the surface model for A_MB_ by quadratic regression revealed that all the coefficients describing the effect of t were non-significant. Therefore, this factor was removed, and the response was described bya second-degree polynomial model depending on T and %NaCl, that was further simplified because also T^2^ and %NaCl^2^ coefficients were non-significant. The final surface (Figure 3) suggests a progressive increase of the response when both T and %NaCl increase. The observed behavior of MB can be interpreted by the enhanced release of this compound from the liquid sample to the headspace, and then to the sorbent, promoted by temperature rising, and by the decrease of its solubility in the wine sample determined by the increase of ionic strength when NaCl is added [16]. 

Different from the behavior of MB, the total area of the chromatogram and, at high ionic strength, that of ED exhibit a noticeable decrease when temperature rises. In addition, a moderate salting out effect seems to only influence A_TOT_ at low %NaCl values (roughly in the range 10–20%), whereas global extraction efficiency slightly decreases at higher salt concentrations. A qualitatively similar effect is observed for A_ED_ at low and intermediate temperatures, whereas, at higher temperatures, the extraction efficiency of ED monotonically decreases when the salt content grows. The increase in temperature, as previously discussed, increases the concentration of the volatiles in the headspace, but partition between headspace and sorbent, which is an exothermic process, is reduced at higher temperature and, consequently, the extraction can be inhibited [16]. In the case of ED, the latter effect seems to prevail on the first, as already documented for several ethyl esters in raw spirits and model wine samples, including ED [30,31]. Since the esters are the volatile components contributing the most to the wine volatilome, it is reasonable that the temperature effects on A_ED_ and of A_TOT_ are qualitatively similar. Regarding the influence of the added salt, in agreement with previous studies [30,31], salting out is more effective in the extraction of ED at a relatively low NaCl concentration (10–20%). It is not perfectly clear why a further increase in salt concentration inhibits the extraction of ED and other long chain esters. It can be supposed that the increase of ionic strength in the sample favors the co-extraction of matrix components [13,32] that can interfere with the extraction of less volatile compounds. Interferences in the extraction of the less volatile components can also take origin from the competition for the sorbent of the more polar components [30], whose mass transport from the sample to the headspace is enhanced by salting out.

### 2.3. Optimization of HS-SPME on the CW/DVB Fiber

Based on the behavior of the PDMS fiber, as discussed in the previous section, the extraction of MB was not affected by t. We also observed that interaction of t with the other two factors was not significant in the surface models of A_TOT_ and A_ED_, whereas the coefficient of the pure t term was positive in both cases. This means that the shape of the surface of these two responses does not depend on t, although, for a fixed T, %NaCl pair the value of the responses increases when t rises. In light of these findings, an investigation of CW/DVB and DVB/CAR/PDMS fibers was carried out by fixing t at its maximum level (30 min) and by exploring only the influence of %NaCl and T. These factors were varied within two levels and an additional point was considered in the center of the 2^2^ DOE, where three replicate experiments were performed. Table 3 displays the matrix of the experiments for the CW/DVB fiber together with the experimental responses. The comparison of the responses provided by this fiber with those collected with the PDMS sorbent reveals a generally less intense chromatogram in the first case. As can be seen, A_TOT_ variations observed within the various DOE points were comparable to the fluctuations due to random factors, evaluated by the replicate experiments in the central point, except for the point (T = 50 °C, %NaCl = 30), in which A_TOT_ was noticeably greater. Therefore, the trend of A_TOT_ within the experimental domain was evident and the maximum condition for the response was unequivocally identified at T = 50 °C and %NaCl = 30, without the need of developing a response surface model. Concerning the extraction efficiency of ED (A_ED_), T seems to have a negligible or poor effect and the maximum of this response is observed at the lowest %NaCl level. On the contrary, extraction of MB (A_MB_) is inhibited at low NaCl contents and the maximum for its peak area in the GC chromatogram is achieved at the maximum level of both factors (T = 50 °C and %NaCl = 30). In summary, a low NaCl content promotes extraction of ED, whereas the addition of NaCl and the increase in temperature improve that of MB.

### 2.4. Optimization of HS-SPME on the DVB/CAR/PDMS fiber 

**Figure 4:**
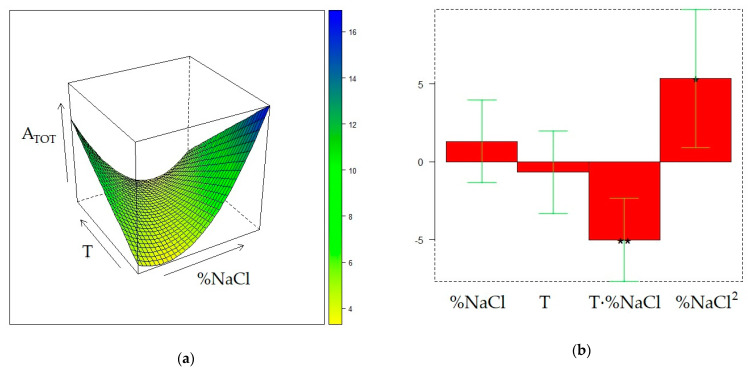
Optimization of HS-SPME profiling of wine volatiles extracted with the DVB/CAR/PDMS fiber: (**a**) response surface for the total area of the GC chromatogram (A_TOT_); (**b**) coefficients of the surface model; *p* < 0.05 (*), *p* < 0.01(**).

**Figure 5:**
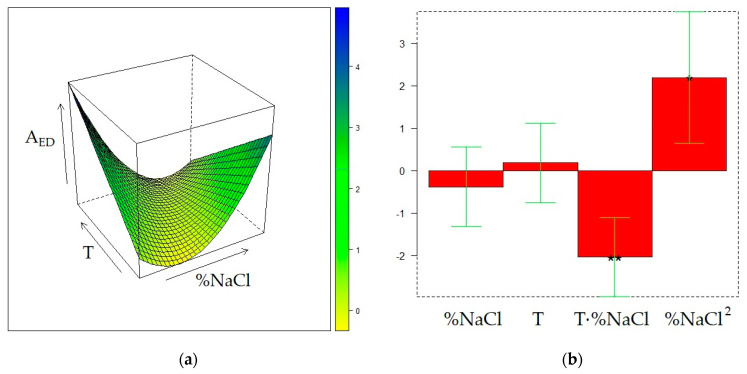
Optimization of HS-SPME profiling of wine volatiles extracted with the DVB/CAR/PDMS fiber: (**a**) response surface for the peak area of ethyl decanoate (A_ED_); (**b**) coefficients of the surface model: *p* < 0.05 (*), *p* < 0.01(**).

**Figure 6:**
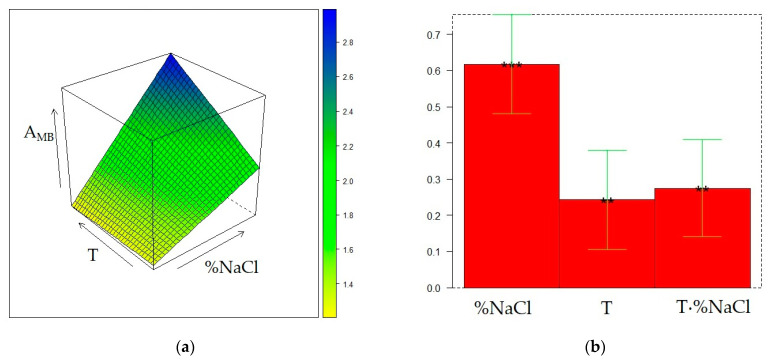
Optimization of HS-SPME collection of wine volatile profiles with the DVB/CAR/PDMS fiber: (**a**) response surface for the peak area of 3-methyl-1-butanol (A_MB_); (**b**) coefficients of the surface model: *p* < 0.01 (**), *p* < 0.001(***).

The same DoE used in the investigation of the CW/DVB sorbent, a 2^2^ one with three additional replicate experiments in the center of the domain, further expanded with replicate experiments in the points (%NaCl = 10, T = 50 °C) and (%NaCl = 30, T = 30 °C), was adopted to model the influence of T and %NaCl on the extraction performance of the DVB/CAR/PDMS fiber. The matrix of the experiments and the experimental and calculated responses are displayed in Table 4. Figure 4, Figure 5, and Figure 6 display the response surfaces and the model coefficients for A_TOT_, A_ED_, and A_MB_, respectively, whereas ANOVA, R^2^_adj_, and Q^2^_adj_ values are reported in Table S2 (Supplementary Material). 

The response surface of A_TOT_ (Figure 4) reveals two maximum conditions, at (%NaCl=10, T = 50 °C) and (%NaCl=30, T = 30 °C), although the value of the response in the latter point is slightly higher than the first. On the other hand, A_TOT_ values in the other DOE points exhibit small differences, comparable with the fluctuations observed in the replicate experiments. The qualitative trend of A_ED_ within the experimental domain (Figure 5) closely reflects that of A_TOT_, although it covers a lower variation range than A_TOT_. It can be also noted that, different from A_TOT_, the maximum in the peak area of ED can be found at the minimum level for %NaCl and at the highest T value. 

It follows that a T increase and a %NaCl decrease result in the increase of the relative contribution of the peak area of ED to the total area of the chromatogram, which agrees with the behavior of ED in SPME conducted with PDMS and CW/DVB fibers, as discussed in the previous sections. The dependence of A_MB_ from T and %NaCl is accurately described by a slightly skewed plane (Figure 6) similar to the surface response observed under the application of PDMS fiber (Figure 3). Furthermore, in this case, despite the variation of this response within the experimental domain being relatively low, the extraction of MB can be enhanced at %NaCl = 30 and T = 50 °C. The same condition was also found as the optimal for the extraction of MB with the CW/DVB fiber. Therefore, whatever the sorbent is, the increase in volatility and salting out, respectively promoted by T and %NaCl rising, improve the MB extraction. On the other hand, the two almost equivalent maximum conditions for A_TOT_ and A_ED_ seem to be originated by antagonist T and %NaCl effects combined with the strong interaction of these two factors. 

**Figure 7:**
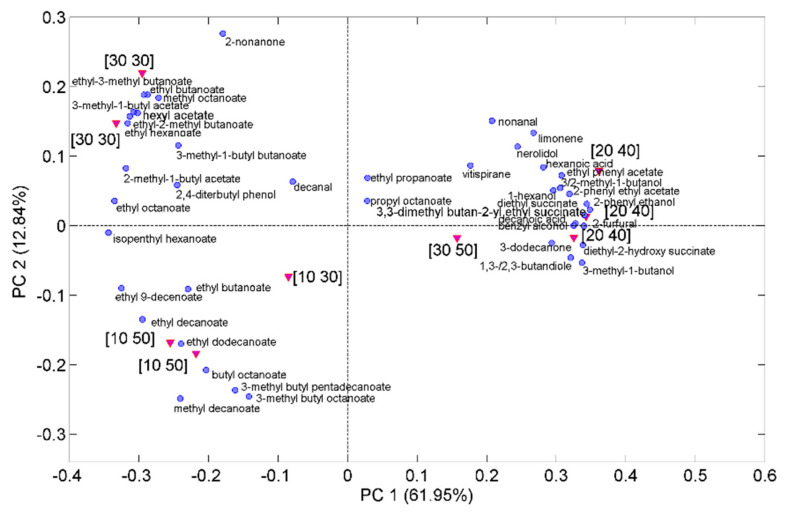
Scores (red triangles) and loadings (blue circles) projected on the plane of the first two principal components (PC1 and PC2) extracted from the GC chromatograms collected with the DVB/CAR/PDMS fiber in the DOE points. Experimental conditions of the DOE points are identified by %NaCl and T values reported in square brackets.

To investigate the influence of the SPME conditions on the volatile profile determined by HS-SPME/GC-MS in more detail, PCA was performed on all the peak areas identified in the chromatograms collected in the various DoE points. Before, each peak area was divided by A_TOT_ and auto-scaling was applied to the normalized quantities with the scope of exalting the role of variables with low variance. Figure 7 displays the DoE points projected onto the plane of the first two principal components (PC1 and PC2, accounting for about 62 and 13% of the total variance, respectively) together with the variable loadings. It can be seen that PC1 describes the greater variation in the volatile composition detected by HS-SPME/GC-MS within the experimental domain, which occurs when we move from the two maxima of the response surface, (%NaCl = 10, T = 50 °C) and (%NaCl = 30, T = 30 °C) located at negative scores, towards the minimum region (represented by the central point and the other two points of the 2^2^ DoE), mainly located at positive score values. Most of the volatile components with positive loadings are relatively polar compounds (alcohols, organic acids, and low molecular weight esters) including MB. On the other hand, the greater values of the A_TOT_ response, identified by negative scores on PC1, are linked to a preferential extraction of the esters. Interestingly, the two maximum conditions, which are well separated along PC2, can be differentiated according to the kind of esters preferentially extracted. More specifically, higher molecular weight esters are preferably extracted at %NaCl = 10 and T = 50 °C, whereas extraction of low molecular weight esters is favored at %NaCl = 30 and T =30 °C. It seems that extraction of polar compounds, which is exalted at the intermediate and highest levels of both T and %NaCl factors, is favored by a salting out effect combined with the easier volatilization of these compounds at higher temperatures, both effects promoting partitioning of the volatiles into the fiber sorbent. Roughly, the distribution of the esters along PC2 follows the inverse order of volatility: long chain esters with higher boiling points have negative loadings, while lower molecular weight and more volatile esters have positive loadings. In this regard, previous investigations regarding the PDMS fiber [31] showed that salting out is more effective for volatile esters, whereas the positive effect of salting is limited to low added concentrations or produces a negative effect as volatility of the esters decreases. As concerning the effect of temperature, a previous investigation regarding extraction of wine components by SPME on PDMS and DVB/CAR/PDMS fibers [31] has highlighted that the increase of temperature in the range 30–60 °C produces a monotonic decrease in the extraction of the most volatile esters, a progressive increase for the less volatile ones, whereas the semi-volatile compounds exhibit a maximum in the extraction efficiency at intermediate temperatures. It is also known that less volatile esters need a longer exposure time to achieve the equilibrium between the sample and the sorbent [30,31] compared to more volatile compounds. It is likely that an exposure time of 30 min, selected in this study, was insufficient to have equilibration of the less volatile esters at the lower temperature level here investigated. The combination of the above effects can explain the preferential extraction of less volatile esters, including ED, at high temperature and low NaCl content and the preferential extraction of more volatile esters at low temperature and high salt concentration.

### 2.5. Aroma Profiles of Trebbiano d’Abruzzo and Pecorino White Wines

**Figure 8:**
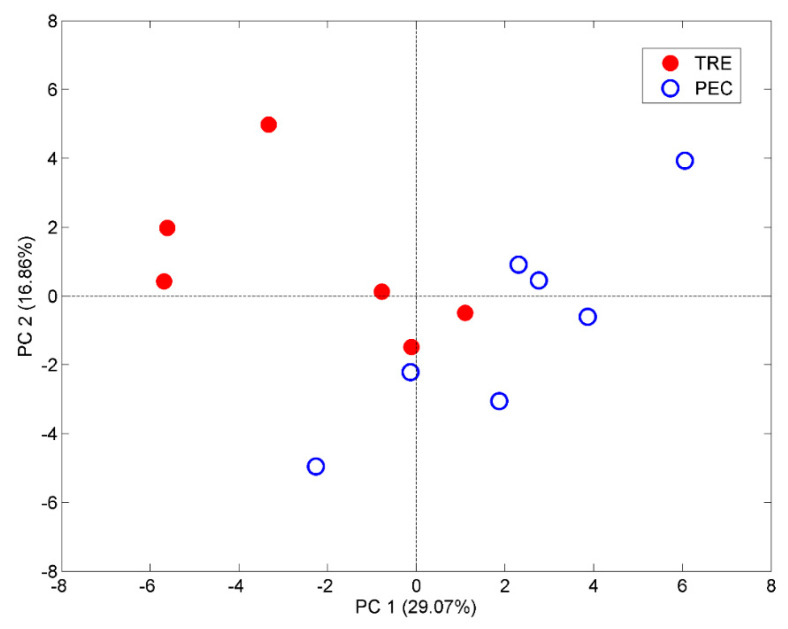
Projection of PEC and TRE wine samples onto the plane of the first two principal components (PC1 and PC2) extracted from the volatile profiles collected with the DVB/CAR/PDMS fiber.

**Figure 9:**
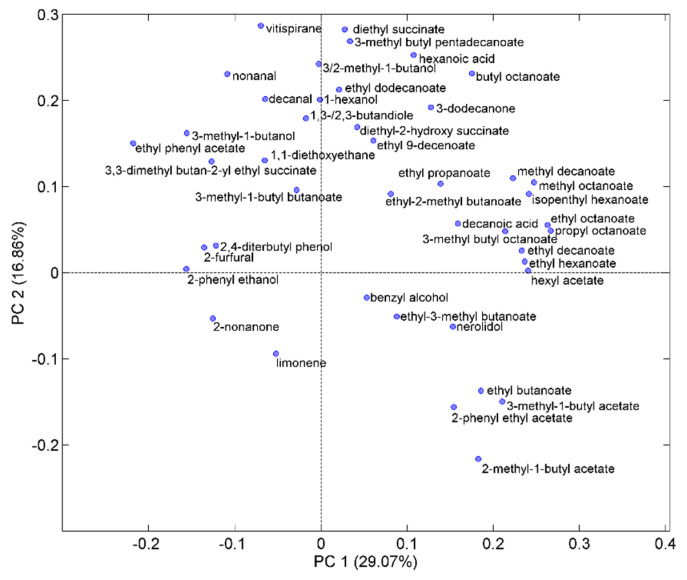
Projection of the variable loadings onto the plane of the first two principal components (PC1 and PC2) extracted from the volatile profiles of PEC and TRE wines collected with the DVB/CAR/PDMS fiber.

Seven PEC and six TRE wine samples were analyzed by HS-SPME GC/MS using the DVB/CAR/PDMS at t = 30 min, T = 30 °C, and %NaCl = 30. This fiber was preferred because, as discussed above, it permits the extraction of a greater number of volatile compounds, compared to PDMS and CW/DVB. In addition, the selected SPME conditions ensure the maximum global intensity of the chromatogram combined with a well-balanced contribution of major and minor components of the wine volatilome. The mean area values (%) of the detected volatiles in the GC chromatograms under the above conditions are listed in Table 1A, together with the related standard errors. A point deserving attention is the potential influence of ethanol on the enrichment of the volatiles on the SPME fiber. Ethanol can compete with the volatile components for the fiber active sites and displace the other compounds in the absorption/adsorption step. A previous investigation on DVB/CAR/PDMS and PDMS sorbents applied to the extraction of representative volatiles in the range of ethanol content between 5 and 90% (*v/v*) [31] has revealed that the extraction effectiveness of volatiles due to the ethanol competition decreases at alcohol contents higher than 20% (*v/v*). Since the wines here analyzed have a lower alcohol content (between 12 and 13.5 %), we can neglect this kind of interference. To investigate the relationship between the aroma composition and wine variety, PCA was performed on the relative areas (%) of the volatile components detected in the TRE and PEC samples. Prior to PCA, the variables were autoscaled to exalt the role of volatile components with low variance. PCA results are reported in Figure 8 (score plot) and Figure 9 (loading plot). Projection of the wine samples into the PC1-PC2 plane (Figure 8) reveals a separation, although incomplete, of the two wine varieties along the bisector of the second and forth quadrants. Acetates, butanoates, nonanal, decanal, and vitispirane are among the volatile components that mostly contribute to the partial differentiation of PEC and TRE wines. On the other hand, variability in the relative content of the major esters, having positive loadings on both PC1 and PC2, seems to be responsible for the relatively large variability in the aroma composition internal to the PEC wine group.

Preliminary PCA of the volatile profiles of TRE and PEC wine samples reveals that the two wine varieties, although produced with grapes belonging to the same family, exhibit a different composition of the aroma profile. Nevertheless, because of the relatively small number of wine samples analyzed here and the explorative nature of PCA, the ability of the volatile profile for the discrimination of mono-varietal wines produced with TRE and PEC grapes has not been demonstrated. The successive development of the present investigation [28], extended to a larger number of TRE and PEC samples representing the four Abruzzo provinces, and including a third wine variety belonging to the family of *Trebbiano* grapes, has shown that the aroma profile alone is not capable of providing an acceptable discrimination of the wines on the varietal basis, while varietal classification improves if the volatile profile is combined with the profiles of organic acids and polyphenols and multi-elemental composition.

## 3. Materials and Methods

### 3.1. Wine Samples and Chemicals 

Commercial bottled wines (2015 vintage) of TRE and PEC varieties, with an alcohol content ranging from 12.0% to 13.5% (*v/v*), produced in various sites of the Abruzzo region, were analyzed. To avoid alteration of wine sensorial properties due to aging, the analysis of the aroma profile was carried out on freshly opened bottles from February to March in 2016. Sodium chloride (purity >99.5%) and Retention Index Standard (aliphatic C_7_–C_24_ hydrocarbons dissolved in hexane) were purchased from Sigma-Aldrich (Saint Louis, MO, USA). Three fibers (Supelco, Bellafonte, PA, USA) coated with different stationary phases and film thickness were investigated: polydimethylsiloxane (PDMS) 100 µm, Carbowax/divinylbenzene (CW/DVB) 65 µm, and divinylbenzene/Carboxen/polydimethylsiloxane (DVB/CAR/PDMS) 50/30 µm. 

### 3.2. Headspace Solid-Phase Extraction of Volatiles

Aliquots of 5 mL of wine samples and NaCl (between 10 and 30% *w/v*) were introduced into a 10 mL vial tightly capped with a PTFE-silicon septum. The sample, kept under magnetic stirring, was heated at constant temperature (within 30–50 °C) using an aluminum block Vertex within 1 °C. Prior to SPME, each fiber was conditioned at the temperature recommended by the manufacturer. The fiber was then exposed to the sample headspace for a fixed time, successively removed, and inserted into the GC injection port where desorption took place at 250 °C for 5 min. 

### 3.3. Chromatographic Analysis

All analyses were carried out on a Varian Saturn 2000 (Varian, Inc., Walnut Creek, CA, USA) GC-MS system composed by a Star GC 3400 CX (Varian, Inc.) gas chromatograph connected to an ion-trap mass detector. The GC apparatus was equipped with a 1078 split/splitless injector with a SPME liner inside. All injections were performed in a split mode with a 50:1 split ratio. A Varian FactorFourTM VF5-ms (Varian, Inc.) capillary column (30 m × 0.25 mm × 0.25 µm film thickness) was used and the carrier gas was Helium IP, supplied at a flow rate of 1.0 mL/min. The column oven temperature program was the following: initial temperature 35 °C for 5 min, then raised at 5 °C/min to 150 °C and held for 1 min, and finally increased to 280 °C with at a rate of 10 °/min and held for 5 min. Retention indices of the extracted compounds were determined on the basis of the observed retention times of aliphatic hydrocarbons (C_7_-C_24_). These data were collected by HS-SPME/GC-MS analysis of the Retention Index Standard after dilution with a water-ethanol mixture, under application of the same temperature program used in the analysis of wine volatiles. 

### 3.4. Design of the Experiments and Response Surface Methodology 

The influence of temperature (T), percentage (*w/v*) of NaCl (%NaCl), and time exposure (t) of the PDMS fiber on the SPME efficiency was investigated using a Box-Behnken DoE. The lowest-highest levels selected for these variables were 30–50 °C (T), 10–30% (%NaCl), and 10–30 min (t), and two replicate measurements were conducted in the central point of the experimental domain. The total area of the GC chromatogram (A_TOT_) was modeled by RSM assuming a polynomial model describing the effects of T, t, and %NaCl (PDMS fiber):

A_TOT_ = a_0_ + a_1_T + a_2_t + a_3_%NaCl + a_12_Tt + a_13_t%NaCl + a_23_t%NaCl + a_11_T^2^ + a_22_t^2^ + a_33_%NaCl^2^ (1)

The response model represented by Equation (1) was also considered to describe the influence of SPME conditions on the peak area of two selected analytes, ethyl dodecanoate (ED) and 3methyl-1-butanol (MB). 

A two-level DoE was adopted to investigate the extraction efficiency of CW/DVB and DVB/CAR/PDMS fibers at t = 30 min by varying T and %NaCl within the same ranges covered in the investigation of the PDMS fiber performance; three additional replicates were carried out in the center of the experimental domain, and duplicate experiments were conducted in two points of the 2^2^ DoE in the case of the DVB/CAR/PDMS sorbent. The response A_i_ (A_TOT_, A_ED_ or A_MB_) was modeled by polynomial regression of the experimental responses against the factors according to the following model:

A_i_ = a_0_ + a_1_T + a_2_%NaCl + a_12_T^.^%NaCl + a_11_T^2^ + a_22_%NaCl^2^ (2)

For each modeled response, possible reduction of the model complexity was evaluated by considering the following hierarchy in the elimination of the non-significant variables: first quadratic, then interaction, and finally, linear terms. Apart from the significance of the model coefficients, the final model complexity was established by evaluating the adjusted determination coefficients in fitting and leave-one-out cross-validation and analysis of variance (ANOVA) to assess the significance of the surface model and lack-of-fit [20]. The response surfaces and models were generated by application of open-source software CAT (Chemometric Agile Tool) [33] freely available on the site of the Italian Group of Chemometrics (http://gruppochemiometria.it/index.php/software, accessed on 13 December 2022).

### 3.5. Principal Component Analysis

Principal Component Analysis (PCA) was applied to evaluate the influence of SPME conditions on the composition of the wine aroma and to make a preliminary comparison of the volatile profiles of TRE and PEC wines. PCA [34] represents multivariate data in a low-dimensionality space of mutually orthogonal, thus uncorrelated, principal components (PCs). They can be defined as the linear combination of original variables explaining unrelated portions of information. Transformation of the original data matrix **X** is described by Equation (3):

**X** = **TP**^T^ + **E** (3)

The loading matrix **P** (with dimension VxA, where V are the original variables and A the number of principal components) defines the new directions. The scores matrix **T** (SxA, where S is the number of samples and A the number of principal components) expresses the coordinates of the samples in the PC space. The error matrix **E** (SxV) collects the residuals associated with the approximation of the original data with fewer PCs than the original variables. To display multivariate information, objects and loadings can be projected onto the compressed PC subspace; this provides a graphical and straightforward visualization of the trends within the data samples (score plot) and interpretation of the selected PCs in terms of the original variables (loading plot). For exploratory analysis, visualization of the data distribution by considering the scores and loadings plot of just the first components (generally two or three) is informative enough, because loss of useful information is generally negligible. PCA was performed using in-house routines in the MATLAB environment (R2019b; The Mathworks, Natick, MA, USA). 

## 4. Conclusions 

In the present work, SPME-based aroma profiling of *Trebbiano d’Abruzzo* and *Pecorino* white wines was optimized through the combination of the DoE approach and RSM modeling of the total area of the GC chromatogram. In addition, to investigate the influence of the SPME conditions, (exposure time of the fiber to the sample headspace, sample temperature and concentration of added salt) on the relative contribution of the volatile components to the collected chromatogram, the peak areas of ethyl decanoate and 3-methyl-1-butanol were also modeled by DoE-RSM. The DVB/CAR/PDMS fiber was identified as the best one, since, compared to PDMS and CW/DVB sorbents, it allowed the extraction of a greater number of volatile components. The response surface of the total area of the chromatogram collected with the DVB/CAR/PDMS stationary phase exhibited two almost equivalent maximum conditions, at an NaCl concentration of 10% and T = 50 °C, and at an NaCl concentration of 30% and T = 30 °C. These maxima are both associated with the efficient extraction of esters from the wine sample. More specifically, the less volatile esters were preferentially extracted under the first experimental conditions, while the more volatile ones were preferentially extracted in the latter conditions. A preliminary comparison of the volatile profiles collected from *Trebbiano d’Abruzzo* and *Pecorino* samples did not provide a clear indication on the possibility of discriminating between these two varieties. Nevertheless, the SPME-based method optimized in the present investigation laid the foundations for a successive data-fusion approach, based on the combination of profiles of volatiles, organic acids, phenolics, and major elements, aimed at the varietal discrimination of wines produced with *Trebbiano d’Abruzzo* grapes and with recently rediscovered autochthone varieties belonging to the same family of grapes, including *Pecorino*. 

## Figures and Tables

**Table 1:** List of the volatile compounds detected (x) in wine aroma by means of different SPME fibers.

Compound	SPME Fiber		
	PDMS	CW/DVB	DVB/CAR/PDMS
ethyl acetate	x	x	-
2-methyl-1-propanol	x	x	x
butanol	-	-	x
ethyl propanoate	x	x	x
1,1-diethoxyethane	-	-	x
3-methyl-1-butanol	x	x	x
2-methyl-1-butanol	x	x	x
ethyl 2-methylpropanoate	x	x	x
1,3-butandiole/2,3-butandiole	x	x	x
1,3-butandiole/2,3-butandiole	x	x	x
ethyl butanoate	x	x	x
ethyl lactate	x	x	x
2-furfural	-	-	x
3-methylbutanoic acid	-	-	x
4-methyl-1-pentanol	-	x	x
ethyl 2-methylbutanoate	x	-	x
ethyl 3-methylbutanoate	x	x	x
1-hexanol	x	x	x
3-methyl-1-butyl-acetate	x	x	x
2-methyl-1-butyl acetate	-	-	x
2-methyl 2,3-pentandiol	-	-	x
4-methyldihydrofurane-2(3H)-one/butyrolactone	-	-	x
anisole	-	-	x
benzaldehyde	-	-	x
hexanoic acid	x	x	x
ethyl hexanoate	x	x	x
hexyl acetate	x	x	x
limonene	-	x	-
2-ethyl hexanol	-	-	x
benzyl alcohol	-	-	x
(E)-2-hexenoic acid	x	-	x
3-methyl-1-butylbutanoate/pentyl 2-methylpropanoate	-	-	x
1-octanol	-	-	x
guaiacol (o-methoxyphenol)	-	-	x
2-nonanone	-	-	x
ethyl eptanoate	x	x	-
lynalol	x	x	x
2-nonen-1-ol/nonanal	-	-	x
2-phenyl ethanol	x	x	x
methyl octanoate	x	x	x
octanoic acid	x	x	-
diethyl succinate	x	x	x
ethyl octanoate	x	x	x
decanal	-	-	x
ethyl phenylacetate	x	x	x
isopenthyl hexanoate	x	x	x
2-phenylethyl acetate	x	x	x
diethyl 2-hydrosuccinate	-	-	x
1-decanol	-	-	x
vitispirane	x	x	x
propyl octanoate	-	-	x
phthalic anhydride	-	x	x
methyl decanoate	-	x	x
butyl octanoate	x	x	x
1,1,6-trimethyl-1,2-dihydronaphtalene	x	x	x
decanoic acid	x	-	x
3-dodecanone	-	x	x
ethyl 9-decenoate	x	x	x
ethyl decanoate	x	x	x
3,3-dimethylbutan-2-yl ethyl succinate	x	x	x
3-methylbutyl octanoate	x	x	x
2,5-di-tert-butylcyclohexa-2,5-diene-1,4-dione	-	-	x
2-dodecenale (E)/dodecanol	-	-	x
2,5-diterbutyl phenol	-	-	x
nerolidol	-	x	x
ethyl dodecanoate	x	x	x
isoamyl decanoate	x	x	-
3-methylbutyl pentadecanoate	-	-	x
galaxolide	-	x	-
2-methylpropyl phthalate	-	x	-

**Table 2:** Points of the Box-Behnken DoE adopted to investigate SPME efficiency of the PDMS fiber with experimental (exp) and calculated (calc) values of the responses: total area of the GC chromatogram (A_TOT_), peak areas of ethyl decanoate (A_ED_), and 3-methyl-1-butanol (A_MB_).

t(min)	NaCl (%)	T(°C)	A_TOT_ 10^−6^		A_ED_∙10^−6^		A_MB_∙10^−6^	
			exp	calc	exp	calc	exp	calc
10	10	40	9.16	8.73	2.66	2.61	0.46	0.49
30	10	40	13.18	12.37	4.98	4.54	0.60	0.49
10	30	40	9.04	9.20	1.47	1.68	1.43	1.40
30	30	40	10.46	10.24	2.24	2.06	1.60	1.40
10	20	30	12.06	11.07	2.99	2.56	0.72	0.70
30	20	50	7.80	7.51	2.46	2.66	1.13	1.19
10	20	50	7.88	7.21	2.37	2.18	1.13	1.19
30	20	30	16.07	16.09	4.42	4.38	0.66	0.70
20	30	50	2.70	2.89	0.16	0.02	1.74	1.77
20	10	30	9.80	10.26	2.41	2.78	0.36	0.37
20	10	50	6.61	7.39	3.34	3.47	0.64	0.60
20	30	30	13.22	13.09	2.71	2.81	0.95	1.03
20	20	40	12.12	12.36	3.18	3.40	0.85	0.95
20	20	40	11.29	12.36	3.15	3.40	0.96	0.95

**Table 3:** Points of the DoE adopted to investigate SPME efficiency of the CW/DVB fiber with experimental values of the responses: total area of the GC chromatogram (A_TOT_), peak areas of ethyl decanoate (A_ED_), and 3-methyl-1-butanol (A_MB_).

NaCl (%)	T(°C)	A_TOT_∙10^−6^	A_ED_∙10^−6^	A_MB_∙10^−6^
10	30	4.30	1.50	0.88
10	50	4.63	1.88	0.94
30	50	9.24	0.38	4.98
30	30	3.95	0.22	2.23
20	40	2.69	0.08	1.19
20	40	4.58	0.28	2.34
20	40	3.86	0.12	2.04

**Table 4:** Points of the DoE adopted to investigate SPME efficiency of the DVB/CAR/PDMS fiber with experimental values of the responses: total area of the GC chromatogram (A_TOT_), peak areas of ethyl decanoate (A_ED_), and 3-methyl-1-butanol (A_MB_).

NaCl (%)	T (°C)	A_TOT_∙10^−6^		A_ED_∙10^−6^		A_MB_∙10^−6^	
		exp	calc	exp	calc	exp	calc
10	30	4.29	4.29	0.52	0.52	1.31	1.27
10	50	12.01	13.02	4.59	4.96	1.30	1.21
10	50	14.02	13.02	5.33	4.96	1.15	1.21
30	30	19.89	16.94	4.87	3.84	1.90	1.96
30	30	13.99	16.94	2.81	3.84	2.05	1.96
30	50	5.57	5.57	0.13	0.13	3.03	2.99
20	40	4.83	4.60	0.15	0.17	1.95	1.85
20	40	4.74	4.60	0.20	0.17	1.78	1.85
20	40	4.24	4.60	0.15	0.17	1.67	1.85

## Data Availability

Not applicable.

## Supplementary Materials

The following supporting information can be downloaded at: https://www.mdpi.com/article/10.3390/molecules28041534/s1, Table S1. Retention time (RT) and experimental (exp) and literature (lit) retention index (RI) of each volatile compound; volatile profiles of PEC and TRE wine samples collected with the DVB/CAR/PDMS fiber under the optimized conditions: mean relative peak area (%) of each compound in the GC chromatogram with the related standard error in brackets. Table S2. Adjusted determination coefficients in fitting (R^2^_adj_) and leave-one-out cross validation (Q^2^_adj_), and ANOVA for the response surface model of the total area of the GC chromatogram (A_TOT_), and peak areas of ethyl decanoate (A_ED_) and 3-methyl-1-butanol (A_MB_) collected with the PDMS and DVB/CAR/PDMS fibers.
