# Supplementary material for: Optimization of HS-SPME-GC/MS Analysis of Wine Volatiles Supported by Chemometrics for the Aroma Profiling of Trebbiano d’Abruzzo and Pecorino White Wines Produced in Abruzzo (Italy)
Source: Molecules. 2023 Feb 5;28(4):1534. doi: 10.3390/molecules28041534 (PMC9962864; doi:10.3390/molecules28041534)
Supplement: Supplementary file 1 [file molecules-28-01534-s001.zip › molecules-2141124-supplementary.pdf]

Table S1. Retention time (RT) and experimental (exp) and literature (lit) retention index (RI) of each volatile compound; volatile profiles of PEC and TRE wine samples collected with the DVB/CAR/PDMS fiber under the optimized conditions: mean relative peak area (%) of each compound in the GC chromatogram with the related standard error in brackets.

RT(min)	RI		compound	Peak area (%)	
	exp	lit		PEC (*n* = 7)	TRE (*n* = 6)
1.99	593	611	ethyl acetate	-	-
2.15	611	625	2-methyl-1-propanol	0.49(0.18)	0.24(0.06)
2.58	647	650	butanol	0.11(0.10)	0.81(0.79)
3.35	705	709	ethyl propanoate	0.31(0.19)	0.028(0.004)
3.74	718	726	1,1-diethoxyethane	0.07(0.03)	0.04(0.02)
3.98	726	736	3-methyl-1-butanol	7.5(0.4)	9.7(1.4)
4.08	730	739	2-methyl-1-butanol	0.29(0.04)	0.60(0.09)
4.65	749	755	ethyl 2-methylpropanoate	0.046(0.005)	0.08(0.02)
5.62	782	788	1,3-butandiole/2,3-butandiole	0.12(0.03)	0.32(0.08)
6.06	797	788	1,3-butandiole/2,3-butandiole	0.024(0.007)	0.051(0.020)
6.22	802	802	ethyl butanoate	0.28(0.03)	0.22(0.05)
6.68	813	815	ethyl lactate	0.18(0.05)	0.50(0.16)
7.50	833	833	2-furfural	0.04(0.02)	0.05(0.02)
7.95	844	863	3-methylbutanoic acid	0.007(0.002)	0.003(0.001)
8.07	847	846	4-methyl-1-pentanol	0.007(0.002)	0.009(0.002)
8.20	850	849	ethyl 2-methylbutanoate	0.013(0.003)	0.017(0.002)
8.41	855	854	ethyl 3-methylbutanoate	0.036(0.004)	0.043(0.004)
9.07	871	868	1-hexanol	0.14(0.02)	0.23(0.03)
9.36	878	876	3-methyl-1-butyl-acetate	2.8(0.2)	2.1(0.3)
9.43	880	880	2-methyl-1-butyl acetate	0.07(0.01)	0.07(0.01)
9.79	889	893	2-methyl 2,3-pentandiol	0.011(0.003)	0.008(0.004)
10.71	911	914/915	4-methyldihydrofurane-2(3H)-one/butyrolactone	0.003(0.002)	0.008(0.003)
10.83	914	920	anisole	0.001(0.001)	0.04(0.04)
12.65	961	962	benzaldehyde	0.21(0.06)	0.2(0.1)
13.40	981	990	hexanoic acid	0.53(0.18)	0.64(0.04)
14.10	999	1000	ethyl hexanoate	6.1(0.2)	6.9(0.6)
14.59	1013	1011	hexyl acetate	0.38(0.06)	0.32(0.07)
15.02	1025	1027	limonene	-	-
15.15	1029	1030	2-ethyl hexanol	0.003(0.002)	0.004(0.002)
15.37	1035	1036	benzyl alcohol	0.06(0.03)	0.04(0.03)
15.72	1045	1045	(E)-2-hexenoic acid	0.016(0.003)	0.027(0.004)
16.11	1056	1056	3-methyl-1-butylbutanoate/pentyl 2-methylpropanoate	0.014(0.002)	0.024(0.004)
16.65	1071	1071	1-octanol	0.019(0.002)	0.031(0.003)
17.04	1082	1085	guaiacol (o-methoxyphenol)	0.0004(0.0004)	0.005(0.003)
17.31	1090	1092	2-nonanone	0.005(0.001)	0.008(0.001)
17.50	1095	1097	ethyl eptanoate	-	-
17.60	1098	1099	lynalol	0.058(0.008)	0.06(0.01)
17.82	1104	1105/1104	2-nonen-1-ol/nonanal	0.03(0.01)	0.08(0.03)
18.05	1112	1116	2-phenyl ethanol	1.9(0.2)	2.9(0.4)
18.41	1123	1126	methyl octanoate	0.035(0.004)	0.034(0.005)
20.00	1172	1180	octanoic acid	--	-
20.19	1178	1182	diethyl succinate	1.7(0.2)	1.9(0.2)

20.72	1195	1196	ethyl octanoate	42.4(0.7)	46(3)
21.04	1205	1206	decanal	0.08(0.04)	0.18(0.06)
22.15	1242	1246	ethyl phenylacetate	0.040(0.008)	0.10(0.02)
22.34	1249	1252	isopenthyl hexanoate	0.092(0.009)	0.10(0.01)
22.51	1254	1258	2-phenylethyl acetate	0.51(0.05)	0.43(0.03)
22.74	1262	1271	diethyl 2-hydrosuccinate	0.033(0.007)	0.04(0.01)
23.02	1273	1270	1-decanol	0.019(0.005)	0.04(0.01)
23.28	1280	1281	vitispirane	0.21(0.04)	0.36(0.08)
23.57	1290	1290	propyl octanoate	0.041(0.004)	0.031(0.004)
24.08	1308	1313	phthalic anhydride	0.025(0.009)	0.05(0.01)
24.48	1322	1325	methyl decanoate	0.031(0.004)	0.05(0.01)
25.13	1345	1348	butyl octanoate	0.022(0.002)	0.026(0.003)
25.40	1355	1354	1,1,6-trimethyl-1,2-dihydronaphtalene	0.10(0.02)	0.16(0.04)
25.71	1366	1373	decanoic acid	0.59(0.05)	0.34(0.10)
26.12	1380	1387	3-dodecanone	0.16(0.04)	0.15(0.03)
26.23	1384	1387	ethyl 9-decenoate	0.83(0.44)	0.65(0.18)
26.45	1392	1396	ethyl decanoate	28.3(0.09)	19.5(1.7)
27.35	1425	1425	3,3-dimethylbutan-2-yl ethyl succinate	0.046(0.006)	0.088(0.008)
27.82	1442	1446	3-methylbutyl octanoate	0.46(0.05)	0.53(0.05)
28.30	1459	1466	2,5-di-tert-butylcyclohexa-2,5-diene-1,4-dione	0.13(0.03)	0.09(0.02)
28.64	1472	1468/1473	2-dodecenale (E)/dodecanol	0.022(0.005)	0.027(0.008)
29.46	1502	1513	2,5-diterbutyl phenol	0.016(0.002)	0.08(0.05)
30.84	1562	1564	nerolidol	0.022(0.004)	0.012(0.005)
31.52	1592	1595	ethyl dodecanoate	1.09(0.17)	0.30(0.14)
32.42	1641	1646	isoamyl decanoate	-	-
32.49	1645	1646	3-methylbutyl pentadecanoate	0.21(0.10)	0.29(0.10)
35.64	1853	1837	galaxolide	-	-
35.74	1861	1870	2-methylpropyl phthalate	-	-

Table S2. Adjusted determination coefficients in fitting (R^2_{adj}) and leave-one-out cross validation (Q^2_{adj}), and ANOVA for the response surface model of the total area of the GC chromatogram (A_{TOT}), and peak areas of ethyl decanoate (A_{ED}) and 3-methyl-1-butanol (A_{MB}) collected with the PDMS and DVB/CAR/PDMS fibers.

Fiber	Response	R^2_{adj}	Q^2_{adj}	Variation source	SS[a]	DF[b]	MS[c]	F-value	Prob-F
PDMS	$A_{TOT} \cdot 10^{-6}$	0.9343	0.8089	Model	139.945	8	17.494	24.092	0.0014
				Residual	3.631	5	0.726		
				Lack of fit	3.286	4	0.822	2.385	0.4474
				Pure error	0.344	1	0.344		
				Total	143.585	13			
PDMS	$A_{ED} \cdot 10^{-6}$	0.8767	0.5625	Model	16.644	8	2.081	12.558	0.0064
				Residual	0.828	5	0.166		
				Lack of fit	0.828	4	0.207	459.961	0.0350
				Pure error	0.0005	1	0.0005		
				Total	17.473	13			
PDMS	$A_{MB} \cdot 10^{-6}$	0.9544	0.9365	Model	2.215	3	0.739	91.639	<0.0001
				Residual	0.081	10	0.008		
				Lack of fit	0.048	5	0.010	1.510	0.3311
				Pure error	0.032	5	0.006		
				Total	2.295	13			

DVB/CAR/PDMS	$A_{TOT} \cdot 10^{-6}$	0.8542	07385	Model	249.529	4	62.382	12.714	0.0152
				Residual	19.627	4	4.907		
				Total	269.156	8			
DVB/CAR/PDMS	$A_{ED} \cdot 10^{-6}$	0.8869	0.7964	Model	40.003	4	10.001	16.687	0.0092
				Residual	2.397	4	0.599		
				Total	42.400	8			
DVB/CAR/PDMS	$A_{MB} \cdot 10^{-6}$	0.9520	0.8669	Model	2.461	3	0.820	53.852	0.0003
				Residual	0.076	5	0.015		
				Lack of fit	0.014	1	0.014	0.891	0.3987
				Pure error	0.062	4	0.016		
				Total	2.537	8			

[a] Sum of squares
[b] Degrees of freedom
[c] Media of the squares
